# Editorial: Pulmonary sarcoidosis

**DOI:** 10.3389/fmed.2023.1177797

**Published:** 2023-03-29

**Authors:** Sahajal Dhooria, Amit Chopra, Mohammed Munavvar

**Affiliations:** ^1^Department of Pulmonary Medicine, Post Graduate Institute of Medical Education and Research (PGIMER), Chandigarh, India; ^2^Division of Pulmonary and Critical Care Medicine, Albany Medical Center, Albany, NY, United States; ^3^Department of Respiratory Medicine, Lancashire Teaching Hospitals, Preston, United Kingdom; ^4^Faculty of Health, University of Central Lancashire, Preston, United Kingdom

**Keywords:** sarcoidosis, diffuse parenchymal lung disease (DPLD), interstitial lung disease (ILD), granuloma, granulomatous disorders

Sarcoidosis is a multisystem granulomatous disorder of unknown cause (1). It is the most common diffuse parenchymal lung disease encountered at most interstitial lung disease centers (2). Though considered a rare disorder, a significant disease burden has been reported from different regions (3). The diagnosis of sarcoidosis rests on finding non-necrotizing granulomatous inflammation in one or more affected organs and excluding its known causes (4). The lungs and intrathoracic lymph nodes are commonly affected; they are also the most frequently accessed organs for diagnostic tissue acquisition (5). A variety of bronchoscopic modalities are used for the same including endobronchial ultrasound (EBUS)-guided transbronchial needle aspiration (TBNA), transesophageal needle aspiration, endobronchial biopsy, transbronchial lung biopsy, and bronchoscopic cryobiopsy (6–10). Recently, the American Thoracic Society has published evidence-based guidelines on sarcoidosis diagnosis (11). The European Respiratory Society and the British Thoracic Society have also published statements on the management of sarcoidosis (12, 13). Most recommendations in these guidelines are based on low-quality evidence. Much research is needed in the diagnostic pathways, treatment, and prognostication of sarcoidosis. We invited manuscripts on various aspects of pulmonary sarcoidosis during a period when the coronavirus 2019 (COVID-19) pandemic was raging. We are pleased to have received contributions from experts including an original article, a systematic review, two narrative reviews, and a case report.

Feng et al. explored the relationship between bronchioloalveolar lavage (BAL) fluid neutrophilia and the severity of pulmonary sarcoidosis in 319 subjects. They found that almost a third of subjects with stage 2 and 3 sarcoidosis had BAL fluid neutrophilia (defined by a BAL fluid differential neutrophil count of >3%). In contrast, a significantly lower proportion of subjects (nearly one in five) with stage 0 and 1 disease exhibited BAL fluid neutrophilia. Disease relapse during follow-up after glucocorticoid treatment was assessed in 123 subjects. BAL fluid neutrophilia was associated with a risk of relapse in a univariate model. The study is an important addition to the scarce knowledge of the predictors of relapse in sarcoidosis. But the study is limited by variable follow-up duration, non-uniform treatment regimens, and lacks granular details of relapses. The authors have also omitted to present a multivariate model, thus restricting any firm conclusions.

Patients with sarcoidosis receiving long-term immunosuppressive treatment might develop COVID-19, which may need a change in therapy. The issue is especially confusing as glucocorticoids are useful in treating acute severe COVID-19 as well as post-COVID-19 persistent inflammatory lung abnormalities (14, 15). There is a suggestion to use the lowest effective glucocorticoid dose and to reduce the intensity of steroid-sparing anti-sarcoidosis (SSAS) agents, especially biologics, when a patient with sarcoidosis develops COVID-19 (16). However, this suggestion is based on expert opinion as there is lack of scientific evidence to support this viewpoint. Kondle et al. performed a systematic search of the literature for reports on COVID-19 treatment of patients with sarcoidosis, who developed COVID-19 disease. They included four case reports and three case series describing 46 patients. Treatment of COVID-19 and adjustment of sarcoidosis therapy were heterogeneous in this group of patients. Some patients continued to receive the same treatment for sarcoidosis that was ongoing before they developed COVID-19, while one or the other immunosuppressive agent (glucocorticoid or the SSAS drug) was tapered or discontinued in others. Interestingly, ongoing biological treatment was discontinued in all six patients once COVID-19 developed. About 13% (6/46) of patients died, suggesting significant mortality. The systematic review does not allow any firm conclusions. After summarizing the available literature, the authors have provided a brief narrative review discussing the use of glucocorticoids, SSAS agents, biologics, and antimalarials. They have also proposed an algorithm for managing sarcoidosis patients who develop COVID-19.

The same group led by Manansala et al. has also contributed a narrative review on COVID-19 vaccination in sarcoidosis. Due to a lack of studies on this topic *per se*, the authors have discussed available information on the seroconversion rates for influenza vaccination in rheumatoid arthritis. The authors conclude that COVID-19 vaccination is safe in sarcoidosis. They propose that patients need to be counseled on the pros and cons of adjusting the immunosuppressive medication in expectation of better COVID-19 vaccine effectiveness. The authors have suggested no adjustment if the patient's current anti-sarcoidosis drugs include glucocorticoids (if dosed at prednisone equivalent < 20 mg/day), antimalarials, leflunomide, azathioprine, and mycophenolate mofetil. They have advised withholding treatment in the “peri-vaccination” period for methotrexate, rituximab, and Janus kinase inhibitors.

We include with great pleasure the narrative review by Judson on granulomatous disorders that resemble sarcoidosis. The article offers a detailed discussion on important sarcoidosis mimics including tuberculosis, chronic beryllium disease, sarcoid-like reaction in solid organ malignancies, granulomatous-lymphocytic interstitial lung disease associated with common variable immune deficiency, and others. The text is supplemented by several radiologic images and two important tables that have listed objectively the features that help distinguish tuberculosis and Crohn's disease from sarcoidosis. The readers will find the information useful in clinical practice. A discussion of modalities that help differentiate sarcoidosis mimics could make the article even more appealing. In this regard, EBUS has emerged as a useful modality that can provide endosonographic imaging and tissue samples from intrathoracic lymph nodes (17, 18). The method has been found to help a great deal in differentiating sarcoidosis, especially from tuberculosis and lymphoma in patients with intrathoracic lymph node enlargement (4, 19, 20). One must also not forget that silicosis and lymphangitis carcinomatosis are close radiologic mimics of sarcoidosis, although they were outside the purview of the review as they are not granulomatous disorders (21).

Finally, we have a case report and review of literature presented by Qiao et al.. The authors report a 30-year-old man with prolonged fever, leg ulcers, splenomegaly, pancytopenia, polyserositis, and lymph node enlargement. Granulomas were found in tissue samples from the lymph nodes, pleura, and leg ulcers. The authors especially excluded tuberculosis and lymphoproliferative disorders by microbiologic examination and bone marrow biopsy, respectively. The patient showed a poor initial response to glucocorticoid treatment but finally became asymptomatic at 9 months. The case is notable for the myriad presentations of sarcoidosis on display. Though limited by the use of inappropriate medical terms in several places, the article commendably provides a thorough review of reports on ulcerative skin lesions, splenomegaly, and serositis in sarcoidosis.

## Author contributions

SD drafted and revised the manuscript. AC and MM revised the manuscript. All authors contributed to the article and approved the submitted version.
